# A Novel Aβ_40_ Assembly at Physiological Concentration

**DOI:** 10.1038/s41598-020-66373-3

**Published:** 2020-06-11

**Authors:** Bogachan Tahirbegi, Alastair J. Magness, Maria Elena Piersimoni, Thomas Knöpfel, Keith R. Willison, David R. Klug, Liming Ying

**Affiliations:** 1 Department of Chemistry, Imperial College London, London, United Kingdom; 2 National Heart and Lung Institute, Imperial College London, London, United Kingdom; 3 Department of Brain Sciences, Imperial College London, London, United Kingdom

**Keywords:** Biophysics, Chemistry

## Abstract

Aggregates of amyloid-β (Aβ) are characteristic of Alzheimer’s disease, but there is no consensus as to either the nature of the toxic molecular complex or the mechanism by which toxic aggregates are produced. We report on a novel feature of amyloid-lipid interactions where discontinuities in the lipid continuum can serve as catalytic centers for a previously unseen microscale aggregation phenomenon. We show that specific lipid membrane conditions rapidly produce long contours of lipid-bound peptide, even at sub-physiological concentrations of Aβ. Using single molecule fluorescence, time-lapse TIRF microscopy and AFM imaging we characterize this phenomenon and identify some exceptional properties of the aggregation pathway which make it a likely contributor to early oligomer and fibril formation, and thus a potential critical mechanism in the etiology of AD. We infer that these amyloidogenic events occur only at areas of high membrane curvature, which suggests a range of possible mechanisms by which accumulated physiological changes may lead to their inception. The speed of the formation is in hours to days, even at 1 nM peptide concentrations. Lipid features of this type may act like an assembly line for monomeric and small oligomeric subunits of Aβ to increase their aggregation states. We conclude that under lipid environmental conditions, where catalytic centers of the observed type are common, key pathological features of AD may arise on a very short timescale under physiological concentration.

## Introduction

A widely adopted framework to understand the biochemical underpinnings of AD is based on the amyloid cascade hypothesis, which assumes that aggregation of the Aβ peptide into oligomeric or fibrous structures triggers a cascade of toxic events that cause functional deterioration of the human brain^1^. The aggregation of Aβ in cell-free solution is well studied and explained by the primary nucleation of monomers, secondary nucleation of monomers on the fibrils and the elongation of fibrils by monomer addition^2,3^. For these molecular events to occur, a critical concentration of monomers of ~90 nM in solution is required^4^, far above the concentration found *in vivo*. Furthermore, for fibril formation in cell free systems, µM concentration of Aβ is required^5^; while in the brain, the peptide concentration reaches only up to 4 nM^6^. Given these numbers it is unlikely that the currently established *in vitro* mechanisms can account for the formation of plaques and tangles in the brain of AD patients^7,8^.

Converse to the aggregation mechanism in the absence of cells, it is well documented that interactions of Aβ with cell membranes can induce Aβ aggregation. Aβ-membrane interactions occur via prion^9^, cholesterol^10^ and gangliosides^11^ or non-specifically with the lipid membranes themselves^12–20^. Previous studies found changes in the lipid composition of the brain in AD patients during disease progression affecting the carbon chain length, linkages, and degree of unsaturation^21^. This brings to the idea that phospholipid metabolism may be an important factor in the pathogenesis of AD^22^. As an amphiphilic peptide, Aβ can interact with multiple lipids and membrane proteins^23^ and the membrane environment also strongly influences the Aβ aggregation^24^. Aβ can disrupt membrane integrity by enhancing reactive oxygen species (ROS) generation and lipid peroxidation causing shortening of lipid carbon chains^23,25–27^ (Supplementary Fig. 1) and increased membrane curvatures^28^. These changes modulate the energetic penalties caused by the hydrophobic mismatch^29–31^, thereby enhancing the interactions between Aβ and membrane. Therefore, it is of great importance to investigate the influence of structure and lipid composition of neuronal membrane on Aβ aggregation and amyloid-induced membrane damage. Indeed, membrane changes may serve as a switch to activate amyloid toxicity^17,32,33^. For example, Aβ has a known ability to bind to cell membranes forming annular oligomers with ring-shaped or pore-like structures with few nanometers in size creating calcium permeable pores in the membranes^34^. This effect allows unregulated entry of Ca^2+^ into neurons^35^. It is unclear if these small oligomers are the toxic species or if there are any species of a size intermediate between oligomers and fibrils inducing Aβ toxicity^36,37^. Here we report a new type of micrometer-size Aβ assemblies associated with lipid membranes.

We studied the formation of Aβ_40_ aggregates on model lipid membranes mimicking healthy (POPC, 16–18 carbon chains, Brain total lipid extracts (BTLE)) and diseased states of neuronal cell membranes^38,39^ (DLPC, 12-carbon chain). We show that the high curvature edges of DLPC membrane patches facilitate specific attachment of Aβ_40_ monomers and small oligomers, forming pearl in a necklace-like structures even when Aβ_40_ concentration is as low as that measured in normal human brains (1 nM). This is the first time that such aggregates have been shown to form within the range of physiological Aβ_40_ concentrations. We also characterized the kinetics of oligomeric structure formation and determined the stoichiometry of small oligomers attached to the edges of membrane patches by employing a variety of fluorescence imaging techniques and atomic force microscopy (AFM).

## Results

### Formation and distribution of giant oligomeric assemblies of Aβ

**Figure 1:**
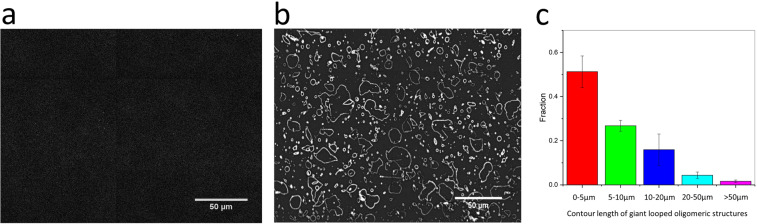
Giant looped Aβ oligomeric structures observed on DLPC membrane by TIRF imaging. (**a**) Before addition of 100 nM Aβ; (**b**) After addition of 100 nM Aβ; (**c**) Contour length distribution of the giant oligomeric structures (N = 1412). Images were analyzed using Imagej (fiji-win64) software (https://imagej.net).

To examine the effect of hydrophobic acyl chain length of the lipids on the aggregation behavior of Aβ, 100 nM dye labelled Aβ was added to different surfaces. These were bare glass, brain total lipid extracts (BTLE), DLPC (12-carbon chain) and POPC (16:18-carbon chain) and the samples were imaged every 2 hours for 48 hours. On DLPC bilayers, spontaneous formation of giant, looped oligomeric assemblies was observed only for the DLPC membrane as shown in Fig. 1a. On plain glass, BTLE and POPC surfaces, no such structures were observed, only heterogeneous deposits of small Aβ oligomers across the planar surface were found. The contour length of giant looped oligomeric assemblies on DLPC varies between 1 to 100 µm (Fig. 1b). Most of these structures are closed.

Giant loops were only observed for DLPC under physiological ionic strength (150 mM NaCl) and was not detected with other lipids e.g. POPC, or natural lipid mixtures BTLE or with DLPC under 100 mM NaCl ionic strength. For these surfaces, only small Aβ oligomers distributed randomly across the surfaces were observed (Supplementary Fig. 2 and Supplementary Video 1).

### Kinetics of Aβ assembly formation

**Figure 2:**
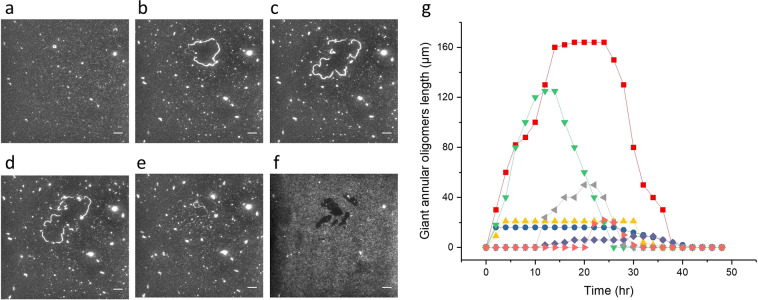
Formation of giant Aβ oligomeric structures on DLPC bilayer during 48 hr. Time lapse fluorescence images of 0 hr (**a**), 6 hr (**b**), 12 hr (**c**), 24 hr (**d**), 36 hr (**e**), 48 hr (**f**); (**g**) Time course of contour length of various oligomeric structures. Scale bar is 10 µm.

The kinetics of the evolution of giant oligomeric assemblies was monitored by time-lapse fluorescence microscopy in the TIRF mode (Fig. 2 and Supplementary Video 2). Adding 100 nM Aβ onto the lipid membrane and monitoring for 48 hours shows the growing and shrinking of these structures (Fig. 2). Such oligomeric assemblies can also be observed by incubation with 1 nM and 10 nM Aβ concentrations (Supplementary Fig. 3a–c).

Giant looped oligomeric structures can grow to different sizes, with the largest size being reached around 12 hr of incubation time (Figs. 1 and 2). Towards the end of recording, [36–48 hrs] large increase of Aβ binding to the DLPC surface was evident (Supplementary Video 2). It is noted that the locations of these structures coincide with the dark patches where their fluorescence is weaker than the neighboring region (Fig. 2f), which is likely due to detachment of lipid membrane over time, leading to increased Aβ deposition in areas where the underlying glass had been exposed. It is known that Aβ molecules have a higher tendency to stick to the lipid membranes that contain cholesterol^40^. We therefore mixed DLPC/cholesterol (molar ratio 85:15) to investigate the effect of the fluidity of the membrane to Aβ assembly. Indeed, addition of cholesterol to DLPC facilitates the attachment of Aβ on top of the membrane patches and allowed us to visualize the patch movement kinetics and the evolution of such giant oligomeric structures over 24 hr with time-lapse fluorescence microscopy in the TIRF mode (Supplementary Video 3). We found that in the presence of cholesterol, Aβ assemblies possess sharper edges and are much less round, indicating that the membrane patches are more rigid than that formed by DLPC alone. This observation suggests that the shape of Aβ assemblies resembles that of the membrane patches and follows the evolution of the local topology of the membrane.

### Location of pearl necklace-like assembly

**Figure 3:**
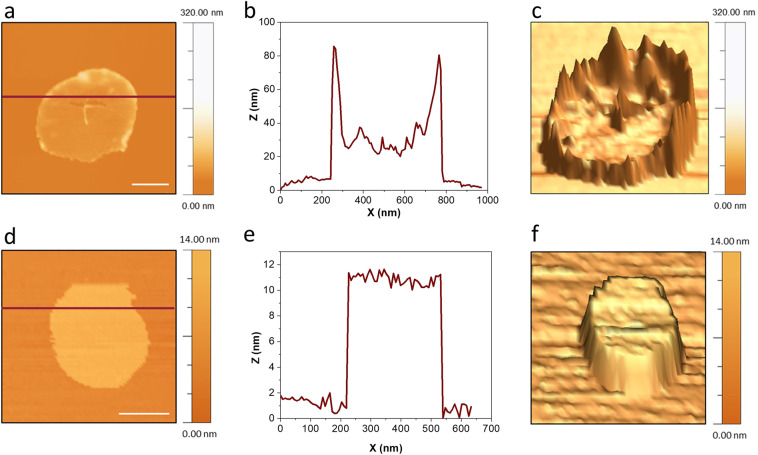
AFM imaging of DLPC lipid patches with and without the attachment of Aβ oligomers. (**a,b,c**) are AFM image, sectional height profile and 3D topography of a lipid patch associated with annular Aβ oligomers. Corresponding AFM image, sectional height profile and 3D topography of a lipid patch without Aβ attachment are shown as (**d,e,f**). All Scale bars are 200 nm.

AFM imaging was performed to confirm if there exist lipid membrane patches whose peripheries preferably attract Aβ to bind (Fig. 3).

Indeed, as illustrated in Fig. 3, oligomers are located at the periphery of lipid patches. The plateau inside the ring-shaped structures allows us to determine the thickness of the lipid patch as ~20 nm. Taking into account that the thickness of a single DLPC bilayer is 5 nm, this membrane patch contains approximately 4 bilayers (Fig. 3). This kind of curved membrane patches has been observed by AFM for different lipids^41,42^. Continuous insertion of Aβ_40_ monomers and small oligomers into the membrane creates closely packed oligomers on the membrane reaching an apparent height of ~80 nm (Fig. 3). As shown in Supplementary Fig. 4, fibrillar structures may start to grow from these ring-like oligomeric assemblies. Remarkably, a long fibrillar structure with a height of ~20 nm seems to be connected to the packed oligomers. The size of this fibril is within the range of the reported dimensions of Aβ mature fibrils^3^. This is in consistent with the suggestion that large oligomers could serve as seeds for fibril growth^43^. Since lipid membrane patches are too fragile to endure time-lapse AFM imaging, we were not able to obtain the time course of fibril growth.

### Super-resolution microscopy and photobleaching experiments at the edges of DLPC membrane patches

**Figure 4:**
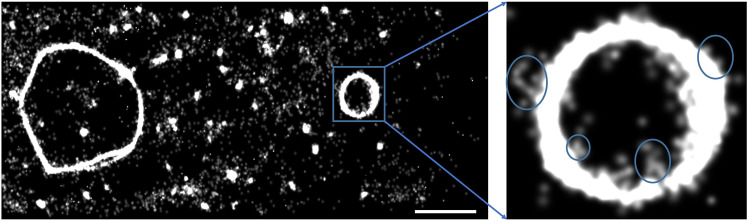
Reconstructed image with Zen Blue software (https://www.zeiss.com/microscopy/int/products/microscope-software/zen-lite.html) by accumulation of single molecules with the superresolution microscope from 10000 frames with 15 ms exposure. The observation of giant looped oligomeric structures with infinite possibilities of interlinked mechanisms for toxic species formation. Scale bar is 2 µm.

**Figure 5:**
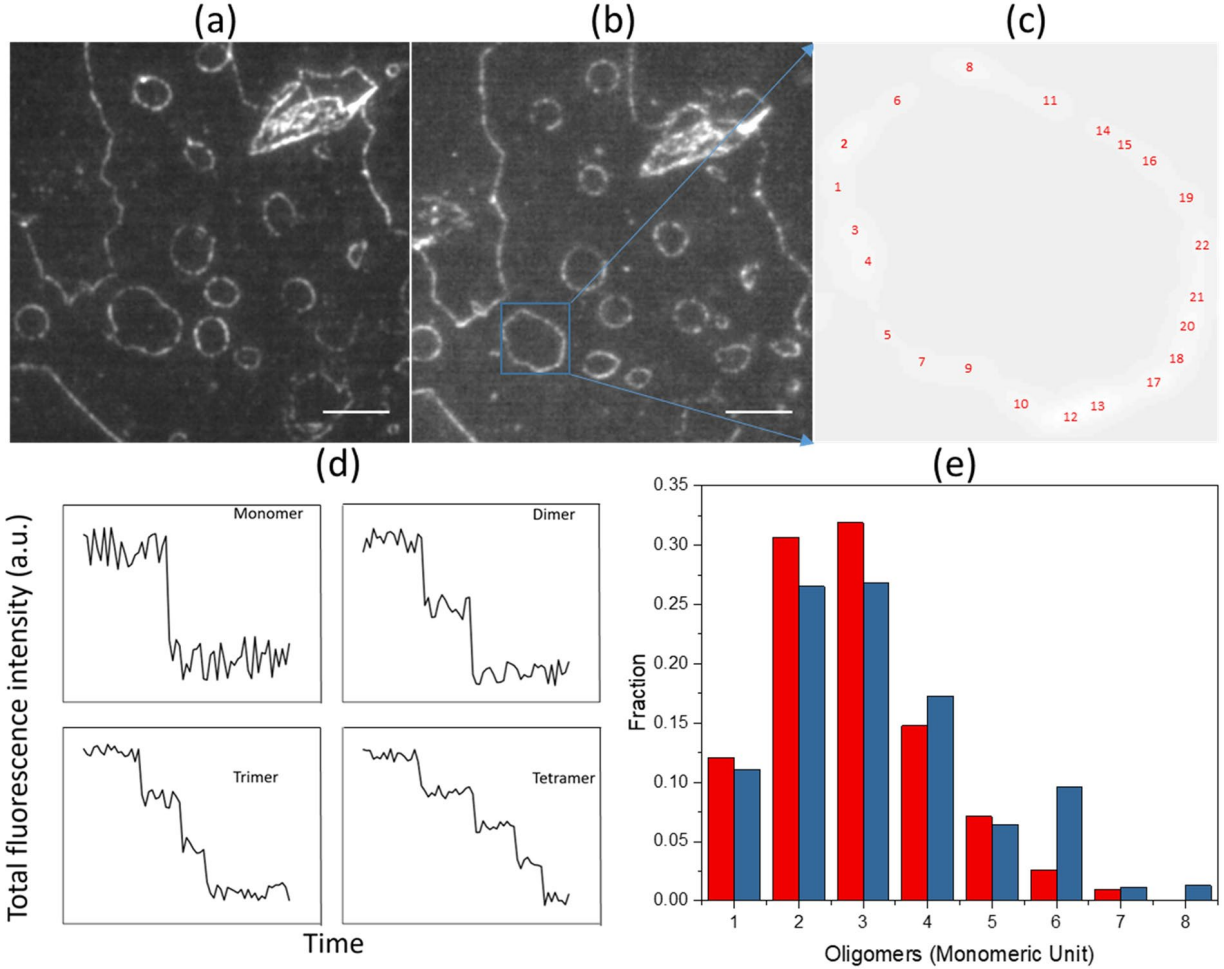
Determination of the size of Aβ oligomers within the pearl necklace-like assemblies. (**a**) Image taken before single molecule photobleaching experiment after 2 hr incubation; (**b**) image taken after complete photobleaching and then another incubation for 12 hr. Scale bars are 10 µm for both **a,b**; (**c**) A localization map for all monomers and oligomer detected in the highlighted assembly; (**d**) Exemplar photobleaching profiles for monomer, dimer, trimer, and tetramer; (**e**) Size distribution of Aβ oligomers with PIF software version 1.1.2 (http://www.biophys.umontreal.ca/bluncklab/software.html) analysis of images taken from 2 hr incubation (Red) and after complete photobleaching and 12 hr incubation for the same areas (Blue).

To investigate whether oligomers on the pearl ring-like assemblies can be exchanged with that from solution, we carried out super-resolution imaging (Fig. 4) and single molecule photobleaching experiments (Fig. 5). A control TIRF imaging experiment was done first to assess the time required for the deposition of Aβ (applied at 100 nM) to the DLPC membrane. As shown in Supplementary Fig. 5, fluorescent intensity of the image reaches maximum after 2 hr incubation. Therefore, superresolution microscopy was performed after an incubation time of 2 hr. Images reconstructed from 10000 images obtained under single molecule imaging conditions (15 ms exposure time) show the continuous deposition and exchange of monomers and small oligomers from solution on top of the edges of DLPC membrane patches. Aβ aggregates, some reminiscent of fibrils, seem to grow from the edges of DLPC membrane patches (Fig. 4). Although many of the molecules are not permanently bound to the DLPC surface, continuous docking and undocking events create a strong background fluorescence. This background fluorescence was eliminated by filtering the data with ZEN software using the localization precision between 1–30 nm and the photon threshold above 200 counts. To prove the existence of preferred region for Aβ attachment, Aβ on the DLPC surface was photobleached while keeping the Aβ concentration in the overlying solution at 100 nM. After all of the oligomers are completely bleached, 10000 images were taken and an image (probability map) was reconstructed from single molecules localizations (Supplementary Fig. 6). The general shape of this reconstruction is almost identical to the one shown in Fig. 4, suggesting that there may be a dynamic equilibrium between monomers and small oligomers in solution and the oligomers on the rings. Supplementary Video 4 illustrates deposition of monomers/small oligomers on the edges of membrane patches.

To further confirm what we observed in the probability map, the same experiment was repeated by analyzing single molecule photobleaching time traces. Labelled Aβ peptide was incubated on DLPC membrane for 2 hr to form the giant looped oligomeric structures. These were photobleached for 3 minutes under 2.5 mW illumination until no fluorescence signal remained (Fig. 5a). After 2 hr incubation, some low order oligomers were formed according to photobleaching analysis (Fig. 5e red). After 12 hr, the same area was imaged by TIRF microscopy and the exact same annular oligomeric structures were observed (Fig. 5b). Although all membrane bound oligomers had been photobleached, the attachment of new monomers on top of the already bound oligomers (Fig. 5b) enabled us to perform a second round of photobleaching. The oligomer size distribution (Fig. 5e blue) was almost identical to that of the previous round (Fig. 5e red). Up to 22 monomer/oligomer spots were resolved by Imagej and PIF software indicating that there are separated oligomers building the giant looped oligomeric structure (Fig. 5c). After buffer exchange to remove Aβ from solution, no further annular oligomer structures were observed 12 hr later. In these experiments, a custom TIRF imaging setup was employed for single molecule photobleaching, which enables us to rapidly extract stoichiometric information about the aggregation state of Aβ oligomers attached to DLPC membrane. The photobleaching data was analyzed with PIF software^44^. Figure 5d shows examples of photobleaching time traces for a monomer, a dimer, a trimer and a tetramer. All the oligomers on the giant looped oligomeric structures in Fig. 5 were analyzed and characterized according to their photobleaching step numbers. Giant looped oligomeric structures were also detected at low nM Aβ concentrations such as 1 and 10 nM (Supplementary Fig. 3).

## Discussion

**Figure 6:**
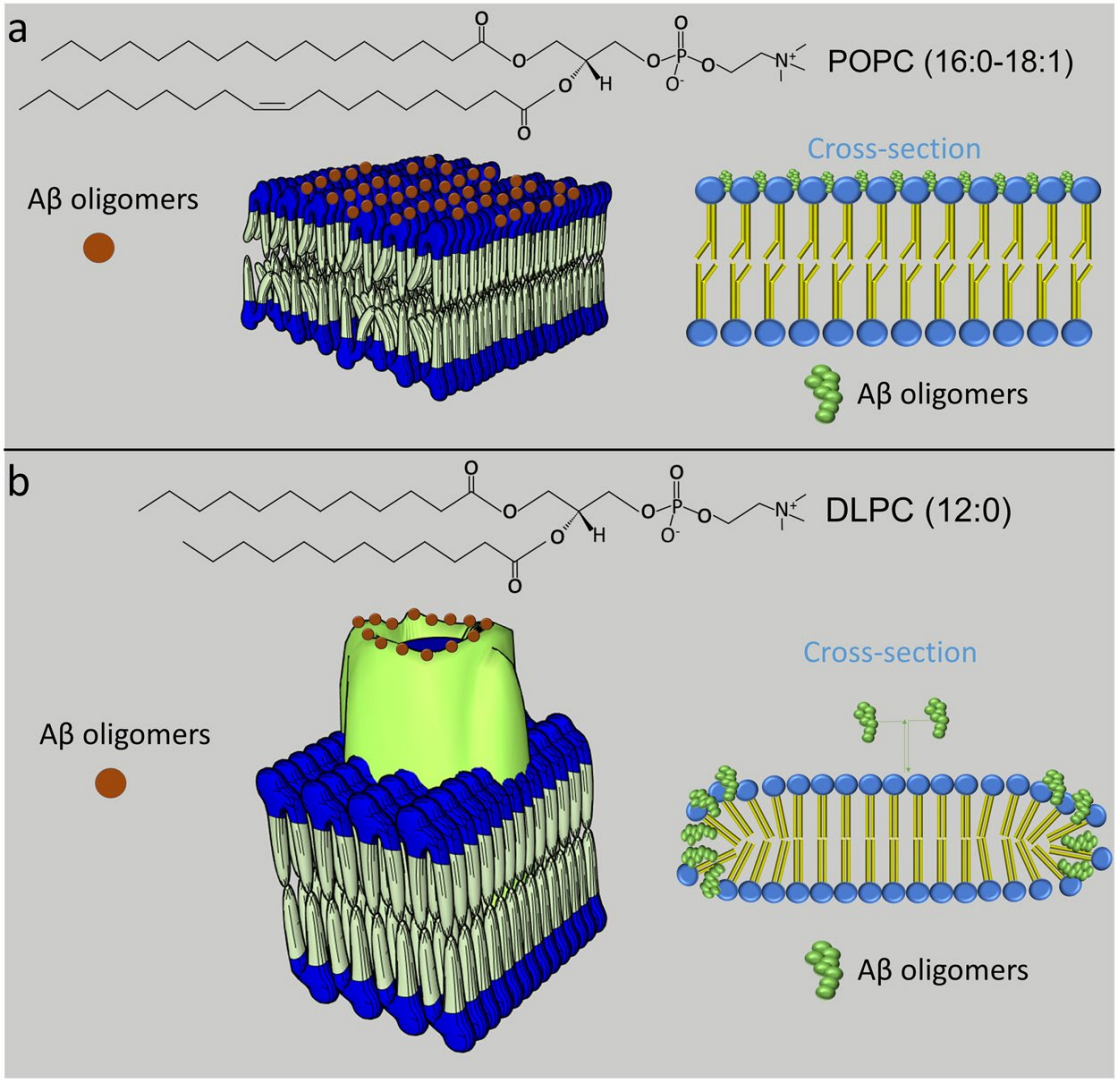
Proposed mechanism of Aβ monomer/oligomer attachment to two different lipid membranes, POPC (**a**) and DLPC (**b**). In this model, Aβ insertion into the membrane is energetically more favourable for DLPC as it relieves the mechanical strain caused by the highly curved regions of the membrane patches, which in turn promotes Aβ aggregation by enhancing its local concentration. The shape of the Aβ assemblies resembles that of the membrane patches. Aβ oligomers coloured in brown interact with defects on the edges of multiple bilayers of DLPC which create hotspots for Aβ attachment.

Local membrane curvature is a key factor defining the morphology of cells, organelles and membrane subdomains. It plays important roles in maintaining trafficking and cellular functions of a living organism^45^. The interactions of intrinsically disordered proteins (IDPs) with curved membrane not only act as a mechanism to sense the membrane curvature^46^ but also promote the crowding and assembly of IDPs themselves (Fig. 6). Such curvature enhanced aggregation has been identified for alpha-synuclein, an IDP linked to Parkinson’s disease and Lewy-body dementia^46,47^. In this study, DLPC membrane was used as a simplified model for thinned, damaged membrane which has a higher propensity to generate membrane curvature (Fig. 6). In the presence of these membranes, giant looped oligomeric structures were observed for the first time at physiological ionic strength and within the range of physiological Aβ concentrations. Both TIRF imaging and AFM imaging show that the pearl necklace-like structures are located at the boundary/edges of membrane patches, resembling their contours (Fig. 6b). In our model, Aβ monomer and small oligomers preferentially bind to the edges of these membrane patches which exhibit higher curvature than the central region lipid bilayer (Fig. 6b). In general, the intercalation of Aβ into regions of high curvature is expected to relieve the bending forces and allow a lowering of the system free energy. It is therefore favorable for Aβ to bind to areas of high curvature.

The assembly of Aβ aggregates orchestrated by a curved membrane involves different forces. This includes the hydrophobic interactions between the lipid acyl chain and the hydrophobic core of Aβ. Hydrophobic mismatch happens when the hydrophobic thickness of the different regions of the membrane varies^29–31^. It is likely that the distinct curvatures that correspond to different hydrophobic mismatches create defects on the edges of multiple bilayers of DLPC and therefore promoting the insertion of Aβ species into the membrane^48,49^. The length of the carbon chain is the most important parameter defining the amphiphilicity of a lipid^50^. It is therefore not surprising that the two model lipids, POPC (Fig. 6a) and DLPC (Fig. 6b), differing by only a few carbon atoms, exhibit huge difference in membrane-catalyzed Aβ aggregation.

This observation suggests that the buildup of shortened lipid and peroxidized lipid, both as a direct result of lipid peroxidation occurring under excess oxidative stress and indirect outcome of impaired repair or cleanup machinery in pathological and ageing conditions, could be a considerable contributor to AD pathology. This assertion is in agreement with the oxidative stress hypothesis of AD^51^.

It is intriguing to note that the number of giant looped oligomeric structures formed depends on the ionic strength. Only at physiological ionic strength (equivalent to ~150 mM NaCl), where the zeta potential of the DLPC lipid bilayer is positive^52^, can the assemblies readily form (Supplementary Fig. 2). At lower ionic strength where the membrane zeta potential is negative, no assemblies were observed (Supplementary Fig. 2).

It should be emphasized that, even at as low as 1 nM concentration of Aβ, the giant looped assemblies of Aβ on DLPC membrane can still be observed to form within a day (Supplementary Fig. 3 and Supplementary Video 5). This concentration is much lower than that required for the formation of oligomers in the absence of membranes, which is 90 nM Aβ^4^. Giant looped oligomeric structures could act as a template for Aβ oligomerization and fibrillation via a variety of pathways and they may even catalyze the secondary nucleation steps (Supplementary Fig. 7 and Supplementary Video 6).

Our results strongly suggest that Aβ aggregates *in vivo* could readily occur on synaptic membrane which has high curvature. The metal ions released during synaptic transmission can induce amyloid-beta oligomer formation in the curved membrane of synapses. Through a combination of experimental kinetics studies and coupled reaction-diffusion simulations, we predicted that Cu (II) rather than Zn (II) plays an important role in the very early stages of Aβ aggregation in the synapse^53^. In addition, curved structures could also take place on cell debris. It is well known that glial cells in the AD brain lose their ability to remove impurities and debris from the brain^54–56^. Some of these may come from multiple lipid bilayers of the myelin sheath debris^55^, mitochondrial membrane or endoplasmic reticulum (ER) of neurons^53^. Aβ aggregation on cell or mitochondrial membrane creates oxidative stress and their interactions with iron and copper generate H_2_O_2_. Interaction of H_2_O_2_ with Fe^2+^ or Cu^+^ generates hydroxyl radical^57^ (OH*), which contributes to the dysfunction of the endoplasmic reticulum (ER) (Supplementary Fig. 1). Therefore, mitochondrial membrane or ER can be a potential source of multiple lipid bilayer debris. Even dead microbes in the AD brain could be a source of membrane fragments. Aβ peptides have been shown to entrap unattached microbes in the brain and have been suggested as a protection mechanism of the immune system^58,59^ in analogue to the functioning of antimicrobial peptides (AMP)^60–62^. It is possible that membrane patches from dead cells either from the body itself or the invaders could provide the right curvature for enhanced Aβ/membrane interaction or therefore assist Aβ assembly.

It should be noted that our current work is limited by the spatiotemporal resolution of super-resolution fluorescence and AFM instrumentation accessible. It would be highly desirable to resolve the time course of Aβ fibril growth from ring-like oligomers which would help unveil the molecular mechanism of Aβ aggregation on damaged cell membrane or membrane patches.

## Methods

### Materials

All reagents were purchased from commercial suppliers and used as received. HiLyte Fluor 647-labeled Aβ40 (HPLC purity >95%) was purchased from Anaspec, which we term Aβ for simplicity in the whole manuscript.1,2-Dilauroyl-sn-glycero-3-phosphocholine (DLPC), brain total lipid extracts (BTLE) and 1–palmitoyl-2-oleoyl phosphatidylcholine (POPC) were purchased from Avanti Polar Lipids Inc. (Alabaster, AL, USA). Methanol (LC-MS grade, ≥99.9%) was provided by Honeywell. 1 M HEPES sodium salt solution, suitable for cell culture and sodium chloride (≥99%) were purchased from Sigma-Aldrich. Lab-Tek Chambered 1.0 Borosilicate cover glass was provided by Thermo Fisher.

### Lipid membrane preparation

10 mg of lyophilized lipid in 1 ml methanol/chloroform was dissolved for a final lipid solution at 10 mg/ml. 100 microliter aliquots of lipid-methanol solution were added to a clean, sterile 2 ml Eppendorf tubes (Final aliquots 1 mg of lipid). Methanol/chloroform from Lipid-methanol aliquots was exposed to a stream of clean nitrogen gas, leaving by evaporation a dry lipid film inside the Eppendorf. To ensure all methanol is removed, eppendorfs were placed inside a vacuum desiccator and degas for overnight. Dried lipid films were stored at −20 °C.

Aliquots were left to warm up to room temperature prior to rehydration. The lipid was then re-suspended in HEPES buffer (50 mM Sodium-HEPES, 100 mM NaCl, pH 7.5.). The solution was pipetted up and down for multiple times until all the lipids are dissolved in the solution. Rehydrated lipid was vortexed and left for 1 hour at room temperature. After 1 hour, the solution was extruded through a polycarbonate membrane which has holes of 100 nm diameter to form small unilamellar vesicles (SUVs). The solution of SUVs was deposited in an 8-well glass slide for fluorescence measurements or in a petri dish with glued borosilicate glass for AFM measurements. Two incubation conditions were tested. In the first one, the glass wells were incubated overnight at 4 °C to allow the vesicles to collapse onto the glass surfaces. This incubation condition is chosen for all the experiments. In the second one, 10 mM Calcium Chloride were added for rapid formation of lipid bilayer and incubated for 30 minutes. This incubation condition is just chosen to limit the formation of multiple bilayers and the limited formation of giant looped oligomeric structures on this surface was tested and compared with the first incubation condition (Supplementary Fig. 3b,c). The glass wells were gently washed with HEPES buffer to remove excess lipid. Aβ_40_ was added to the solutions in the well to a final concentration of 1, 10 or 100 nM respectively.

### Time-lapse TIRF imaging and single molecule photobleaching of annular oligomers

For time-lapse imaging, an objective-type total internal reflection fluorescence (TIRF) microscope based on an inverted Eclipse Ti-E optical microscope (Nikon, Japan) equipped with a 60X NA 1.49 oil immersion objective (model, Nikon, Japan) and an electron-multiplied CCD (IXON DU-897E; Andor Technologies, Ireland) 512 ×512 pixels was used to measure the formation and growth of giant looped oligomeric structures. A diode pumped solid state CW laser (Laser 2000, UK) at 647 nm is coupled into a single-mode optical fiber which is connected to a motorized TIRF illumination unit (Nikon, Japan). The power at the back aperture is adjusted by neutral density filters to 0.7 mW. Chambered cover glass was loaded with 400 µl pure buffer (0.05 M HEPES 0.1 M NaCl, pH 7.4) onto the sample stage of Nikon Ti for control experiments. Different areas inside each chamber were imaged automatically by an encoded high speed XY stage with focal plane being maintained by a Perfect Focus System (PFS) (Nikon, Japan). 1, 10 or 100 nM of Beta-Amyloid (1–40), HiLyte Fluor 647-labeled Aβ sample was added to wells and the formation of giant looped oligomeric structures were recorded every 2 hr for 48 hr. The contour length of each giant looped oligomeric structures was calculated using imageJ software.

For single molecule photobleaching experiments, a custom TIRF imaging setup based on Nikon Eclipse TE2000-U microscope (Nikon, Surrey, U.K.) was used. The laser source was a 633 nm He-Ne laser with 2.5 mW power. The images were recorded by a sCMOS camera (ORCA-Flash 4.0 V3 Digital CMOS camera, Hamamatsu Photonics, Japan). 80 ms recordings with 2 × 2 binning were taken. Fluorescent spots were analyzed by measuring the integrated intensity of individual spots over the course of all 1000 frames. Traces were produced and analyzed by PIF software written by the Blunck group^44^ to automatically analyze single subunit counting data. This software can distinguish individual oligomer spots and analyzes their fluorescence intensity trace for the determination of number of step-wise photobleaching events. It was designed as a fully-automated algorithm that determines the step number distributions, from which the subunit composition can be derived. Thus, large datasets can be analyzed without any user-bias in selection or interpretation of the data. For the analysis of individual molecules, Gaussian blur filter is applied to reduce the effect of imaging noise. We examined the sample quality using single molecule photobleaching analysis to ensure that the percentage of monomeric peptide is always greater than 75%. The rest are small oligomers up to the size of tetramer. Aβ_40_ was chosen for all the experiments since Aβ_42_ has a higher tendency to aggregate and can form hexameric or dodecameric structures in contrast to Aβ_40_ which forms oligomers smaller than hexamers^63^. Furthermore, the fluorescence tag may influence Aβ/membrane interaction and the behaviour of Aβ aggregation. Aβ_40_ with HiLyte Fluor 647 tagged in the N-terminus of the peptide was used to minimize the effect.

### Super-resolution imaging of annular oligomeric assembly

The standard STORM superresolution imaging method achieves increased spatial resolution by sequentially photoswitching individual fluorophores between a fluorescent state and a non-fluorescent state, which leads to a temporal separation of the individual fluorophores from an ensemble of emitters. Basically, it uses the stochastic photo-activation of a permanently bound fluorophore^64^. In contrary, DNA points accumulation for imaging nanoscale topography (DNA-PAINT) uses the stochastic binding of a fluorescent ligand^65^. DNA-PAINT is a quantitative method, because continuous replacement of imager strands from solution avoids photobleaching^65^. We adopted the later principle to image oligomeric annular structures. Continuous replacement and attachment of monomers and small oligomers creates blinking events. Superresolution fluorescence imaging was performed using a Zeiss Elyra PS-1 (Dual- electron-multiplied-CCD iXon PCO Edge sCMOS; Andor Technologies, Ireland) super resolution microscope equipped with an Alpha Plan-APO 100×1.46 NA Oil immersion objective at 640 nm excitation and collecting fluorescence through a 650 nm long pass filter. Image acquisition were carried out under TIRF mode with an excitation laser power density of ~2 kW/cm^2^ and exposure time of 15 ms exposure. 10,000 frames were acquired and single molecule localizations were identified and super-resolution images reconstructed using the Zeiss Zen software. The localization precision used for superresolution image reconstruction is 30 nm.

### AFM imaging

AFM experiments were performed on a NanoWizard 4 BioScience AFM (JPK Instruments, Germany) integrated in an iX81 optical microscope frame (Olympus, Belgium). High-resolution AFM topographical images of lipid bilayers and Aβ oligomers and fibrils were taken using a silicon nitride tip attached to a soft triangular backside gold coated silicon nitride cantilever (MLCT-BIO, cantilever C, Bruker: nominal tip radius of 20 nm, nominal spring constant 0.01 N/m and resonant frequency 7–10 kHz). Force spectroscopy was performed in the Quantitative Imaging mode (QI) of the JPK system (QI-JPK). Lipid bilayers, Aβ oligomers and fibrils were imaged in QI mode at a resolution of 128 × 128 pixels using the sharp cantilever with a set point of 1.5 nN. The images were analyzed by JPK software and WSxM software.

## Supplementary information

Supplementary Video Legends

Supplementary Figure S1

Supplementary Figure S2

Supplementary Figure S3

Supplementary Figure S4

Supplementary Figure S5

Supplementary Figure S6

Supplementary Figure S7

Supplementary Figures

Supplementary Video S1

Supplementary Video S2

Supplementary Video S3

Supplementary Video S4

Supplementary Video S5

Supplementary Video S6
