# Supplementary material for: A Novel Aβ40 Assembly at Physiological Concentration
Source: Sci Rep. 2020 Jun 11;10:9477. doi: 10.1038/s41598-020-66373-3 (PMC7289798; doi:10.1038/s41598-020-66373-3)
Supplement: Supplementary file 1 — Supplementary Video Legends [file 41598_2020_66373_MOESM1_ESM.docx]

**Movie S1.** Time lapse fluorescence images of the attachment of Aβ monomers and small oligomers on BTLE surface for 48 hr.

**Movie S2.** Time lapse fluorescence images of the formation of giant Aβ oligomeric structures on DLPC surface during 48 hr

**Movie S3.** Time-lapse fluorescence images of the patch movement kinetics and the evolution of such giant oligomeric structures for 24 hr on top of cholesterol doped DLPC surface

**Movie S4.** Single molecule localisations by superresolution microscope were accumulated from 10000 frames with 15 ms exposure time illustrating deposition of monomers/small oligomers on the edges of membrane patches.

**Movie S5.** Imaging Aβ assemblies and growth at 1 nM Aβ concentrations on DLPC membranes for 24 hr. The giant looped assemblies of Aβ on DLPC membrane can still be observed to form within a day.

**Movie S6.** Representative reconstruction video of Aβ aggregation processes on the edges of a DLPC membrane patch. Giant annular oligomers could act as a template for Aβ oligomerization and fibrillation via a variety of pathways and they may even catalyze the secondary nucleation steps
