# Supplementary figures and images for: A Novel Aβ40 Assembly at Physiological Concentration
Source: Sci Rep. 2020 Jun 11;10:9477. doi: 10.1038/s41598-020-66373-3 (PMC7289798; doi:10.1038/s41598-020-66373-3)

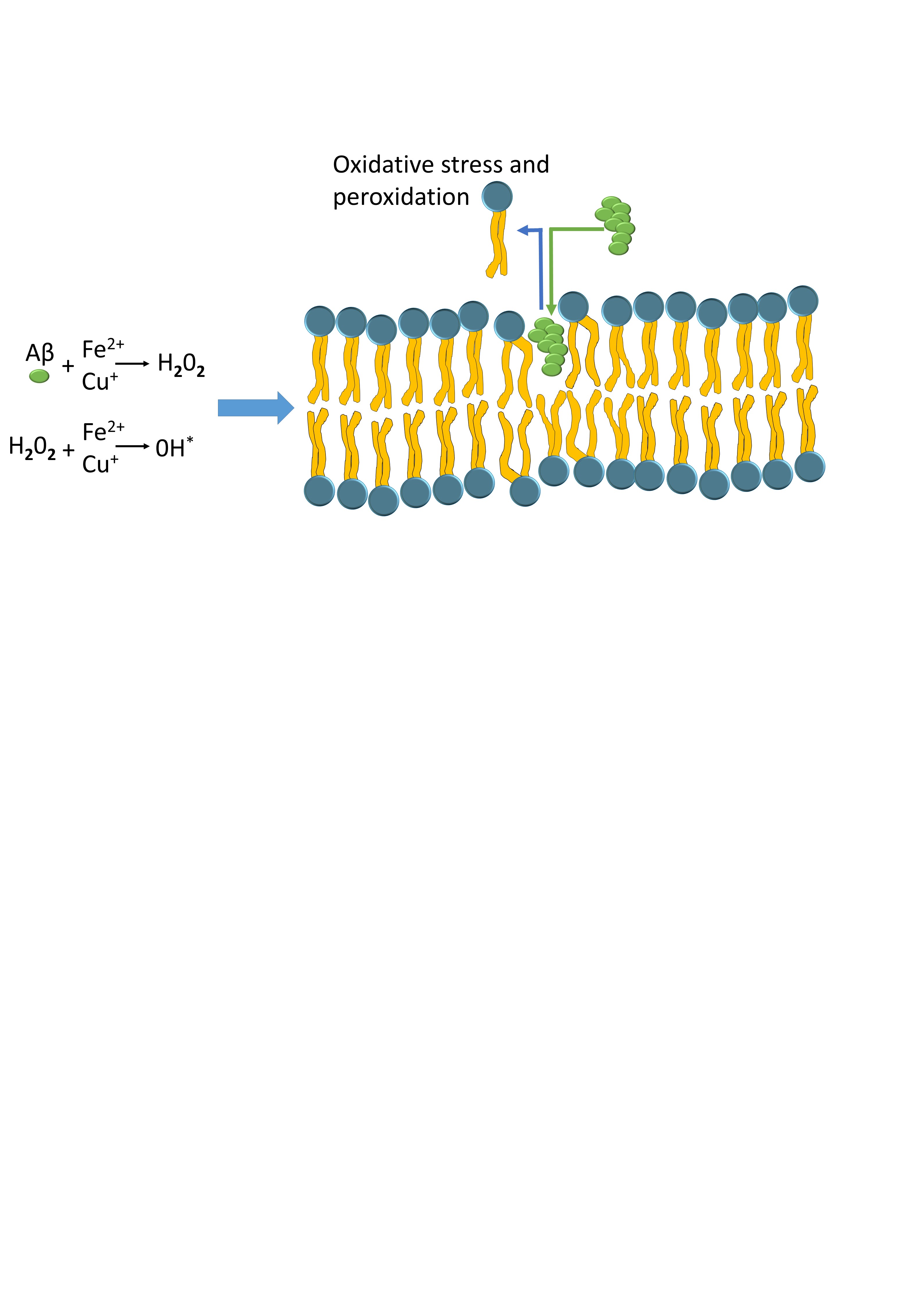

Supplement: Supplementary file 2 — Supplementary Figure S1 [file 41598_2020_66373_MOESM2_ESM.jpg]

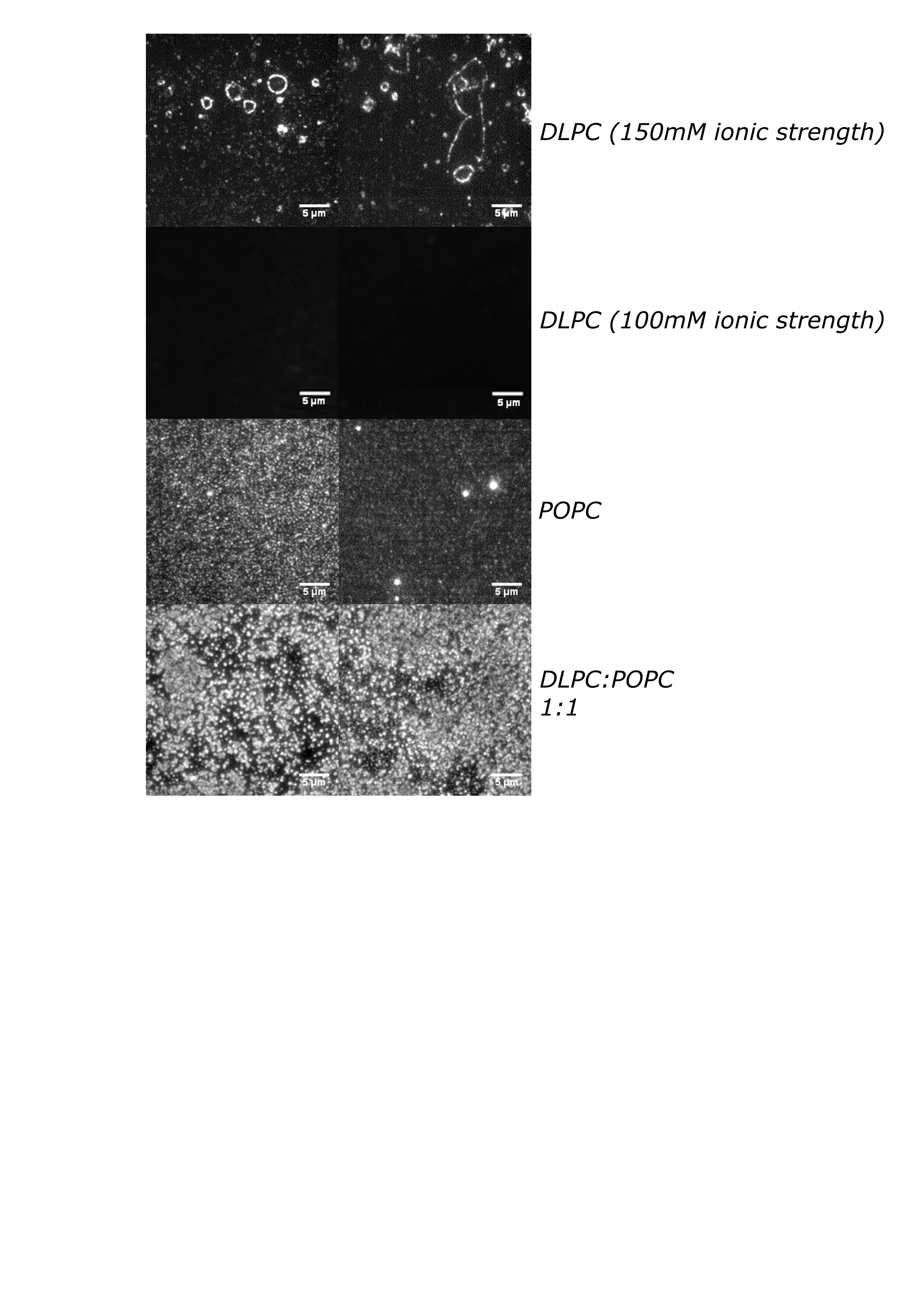

Supplement: Supplementary file 3 — Supplementary Figure S2 [file 41598_2020_66373_MOESM3_ESM.jpg]

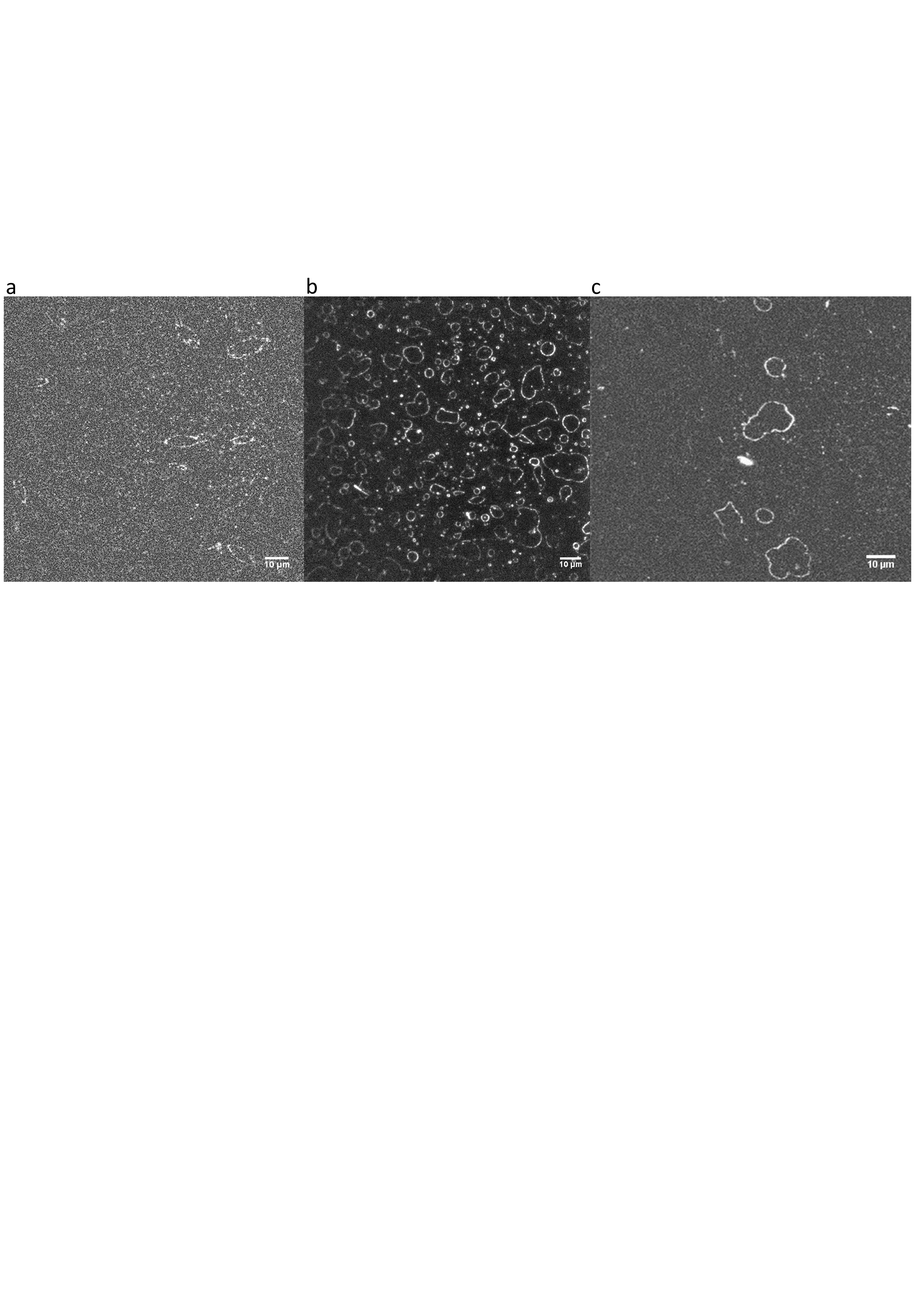

Supplement: Supplementary file 4 — Supplementary Figure S3 [file 41598_2020_66373_MOESM4_ESM.jpg]

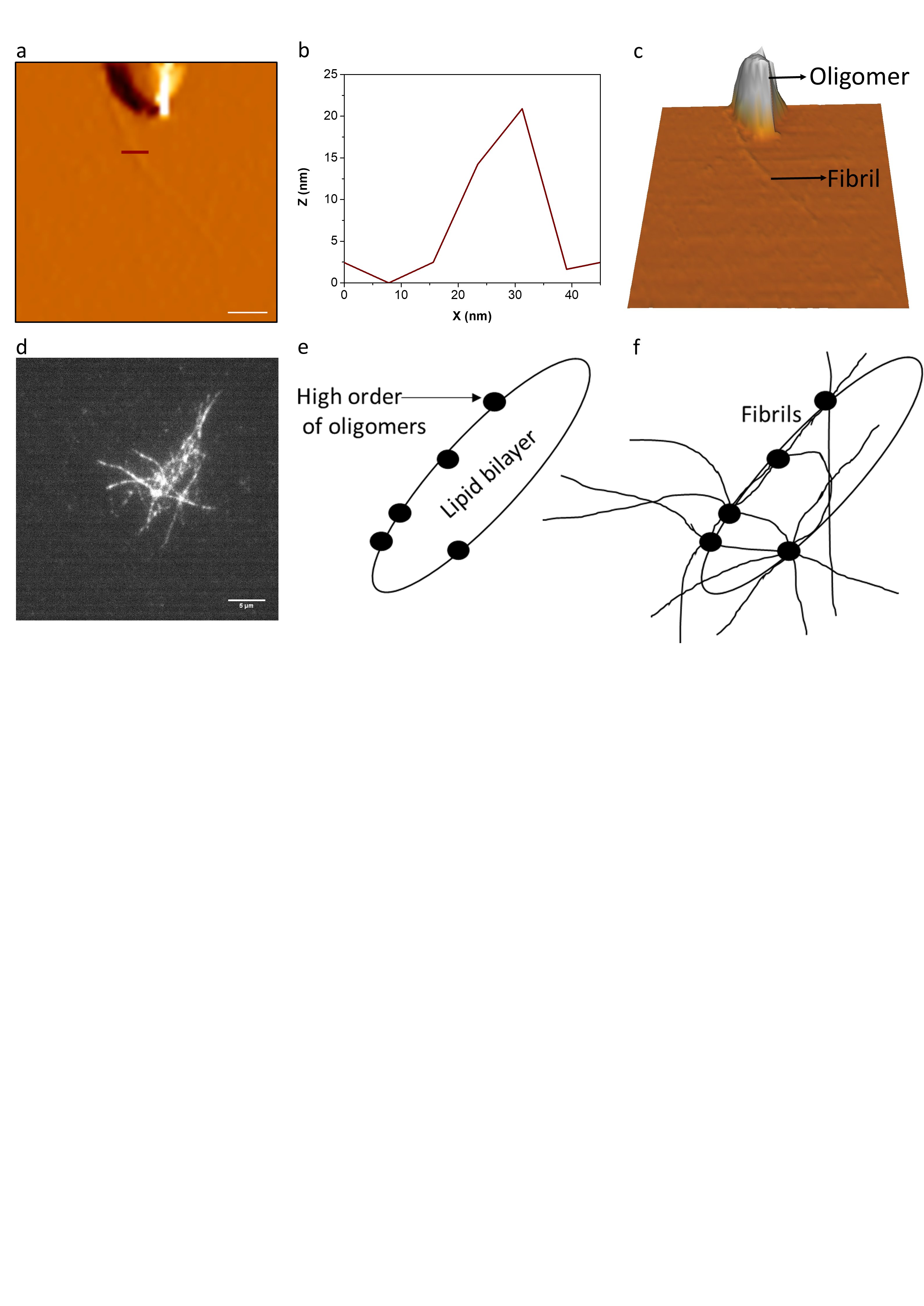

Supplement: Supplementary file 5 — Supplementary Figure S4 [file 41598_2020_66373_MOESM5_ESM.jpg]

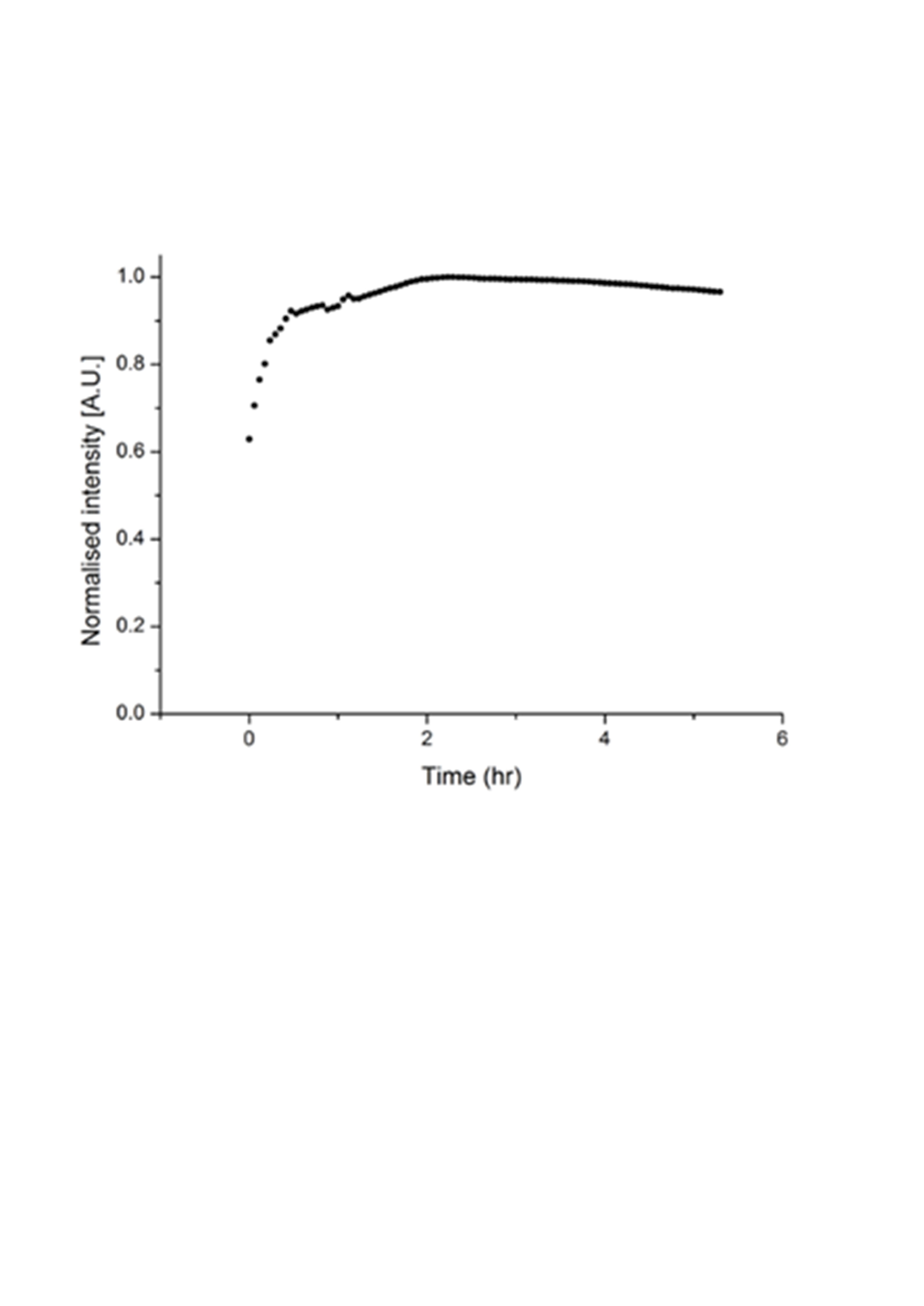

Supplement: Supplementary file 6 — Supplementary Figure S5 [file 41598_2020_66373_MOESM6_ESM.jpg]

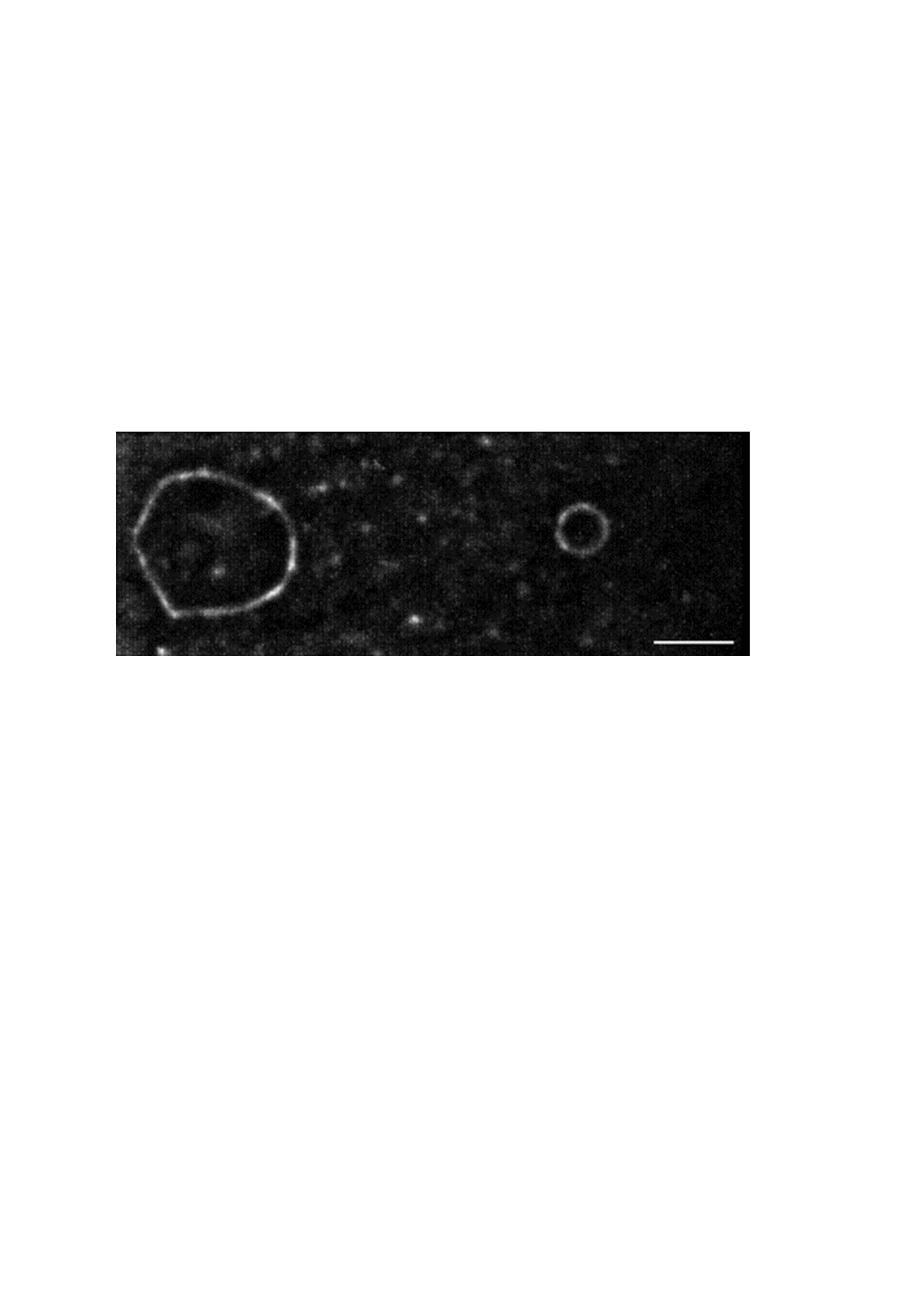

Supplement: Supplementary file 7 — Supplementary Figure S6 [file 41598_2020_66373_MOESM7_ESM.jpg]

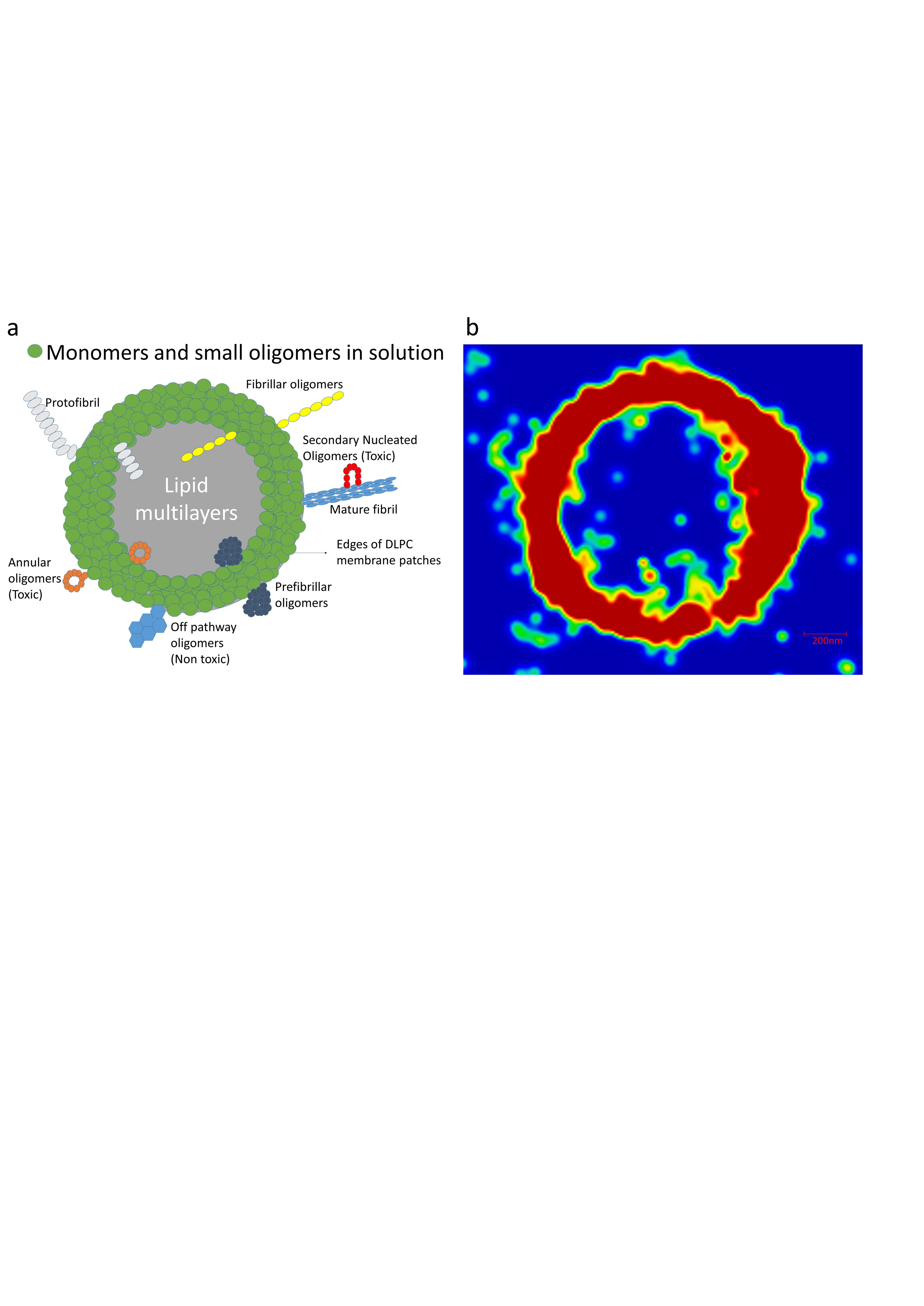

Supplement: Supplementary file 8 — Supplementary Figure S7 [file 41598_2020_66373_MOESM8_ESM.jpg]
