# Supplementary material for: A Novel Aβ40 Assembly at Physiological Concentration
Source: Sci Rep. 2020 Jun 11;10:9477. doi: 10.1038/s41598-020-66373-3 (PMC7289798; doi:10.1038/s41598-020-66373-3)
Supplement: Supplementary file 9 — Supplementary Figures [file 41598_2020_66373_MOESM9_ESM.docx]

**Supplementary information**

**A Novel Aβ_40_ Assembly at Physiological Concentration**

Bogachan Tahirbegi^1,Ϯ^, Alastair J. Magness^2,Ϯ^, Maria Elena Piersimoni^2^, Thomas Knöpfel^3^, Keith R. Willison^1^, David R. Klug^1,*^, Liming Ying^2,*^

^1^Department of Chemistry, Imperial College London, London, United Kingdom

^2^National Heart and Lung Institute, Imperial College London, London, United Kingdom

^3^Department of Brain Sciences, Imperial College London, London, United Kingdom

^Ϯ^These authors contributed equally to this work.

*Correspondence and requests for materials should be addressed to D.R.K. (email: d.klug@imperial.ac.uk), or L.Y. (email: l.ying@imperial.ac.uk).


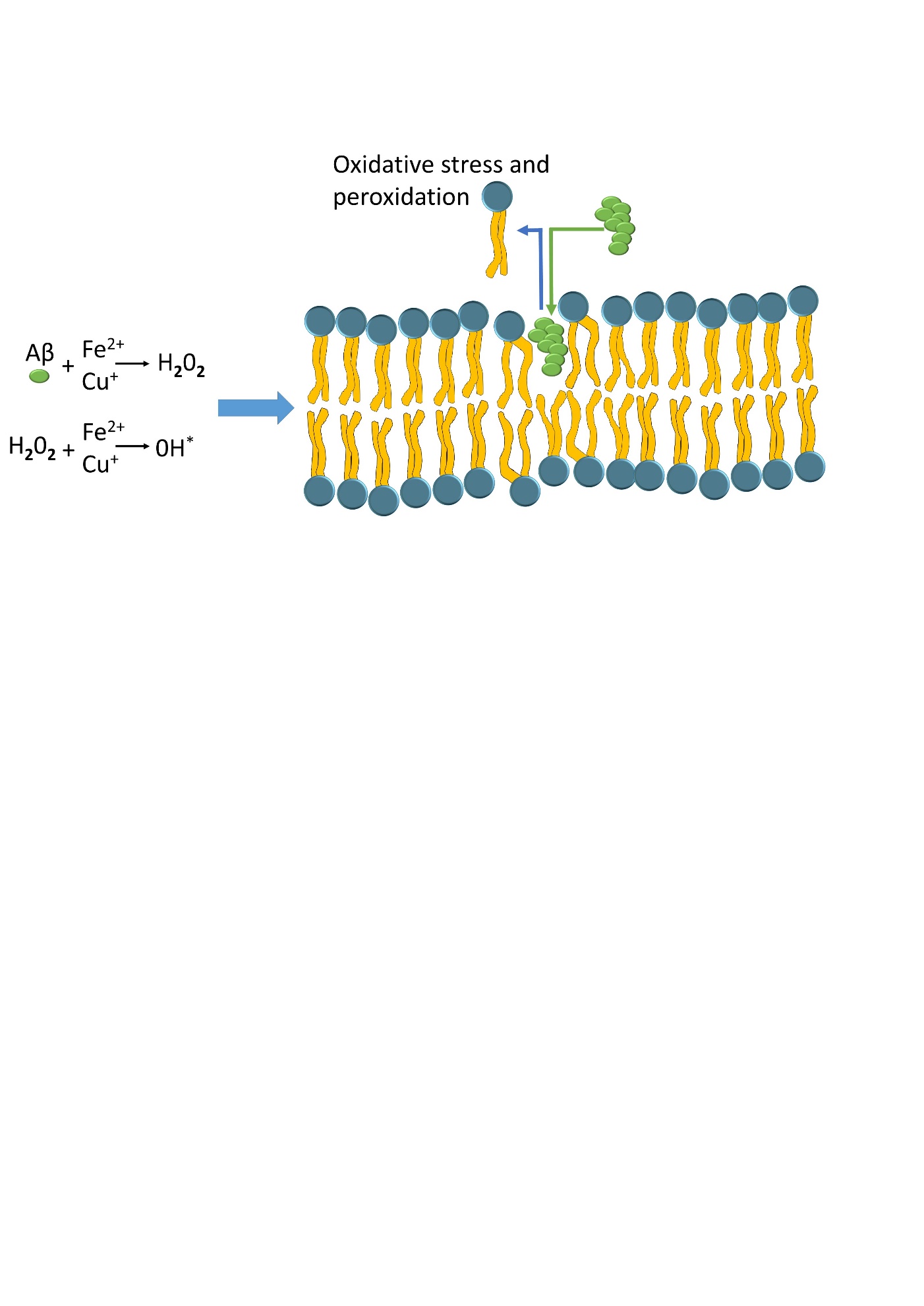


**Supplementary Figure 1: Aβ oligomers on membrane can potentially generate ROS upon interactions with Cu^+^ and Fe^2+^.** Aβ aggregation on cell or mitochondrial membrane creates oxidative stress and their interactions with iron and copper generate H_2_O_2_. Interaction of H_2_O_2_ with Fe^2+^ or Cu^+^ generates hydroxyl radical (OH*), which contributes to the dysfunction of the endoplasmic reticulum (ER). Aβ can disrupt functionalities of membrane by enhancing reactive oxygen species (ROS) generation and lipid peroxidation causing shortening of lipid carbon chains and the formation of membrane curvatures. This modulates energetic penalties caused by the hydrophobic mismatch.


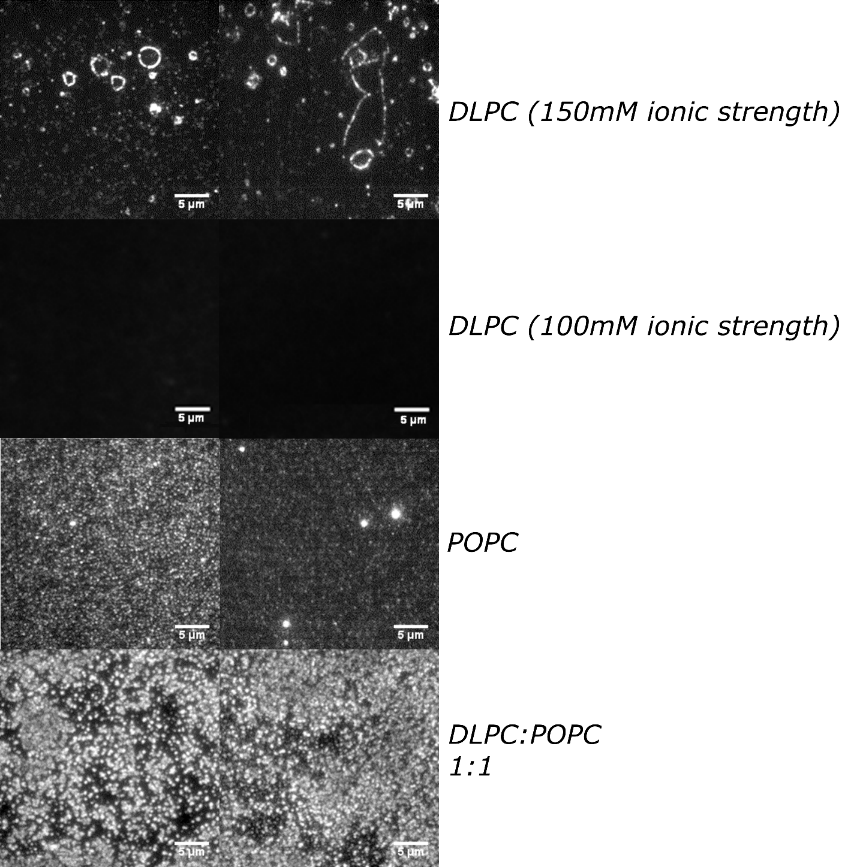


**Supplementary Figure 2: Fluorescence images of 100 nM labelled Aβ on different lipid membranes.** Giant looped oligomeric structures start to occur only for DLPC membrane at 150 mM ionic strength. For the other types of lipid bilayers, Aβ spreads all over the surface.


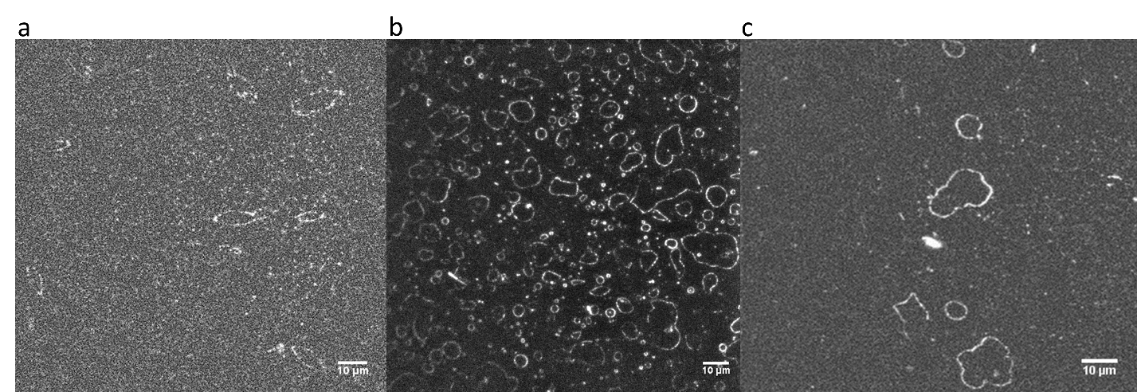


**Supplementary Figure 3: Imaging Aβ assemblies at lower concentrations on DLPC membranes prepared without and with calcium.** (a) 1 nM Aβ; (b) 10 nM Aβ; (c) 10 nM Aβ on the membrane prepared in the presence of 10 mM calcium chloride.


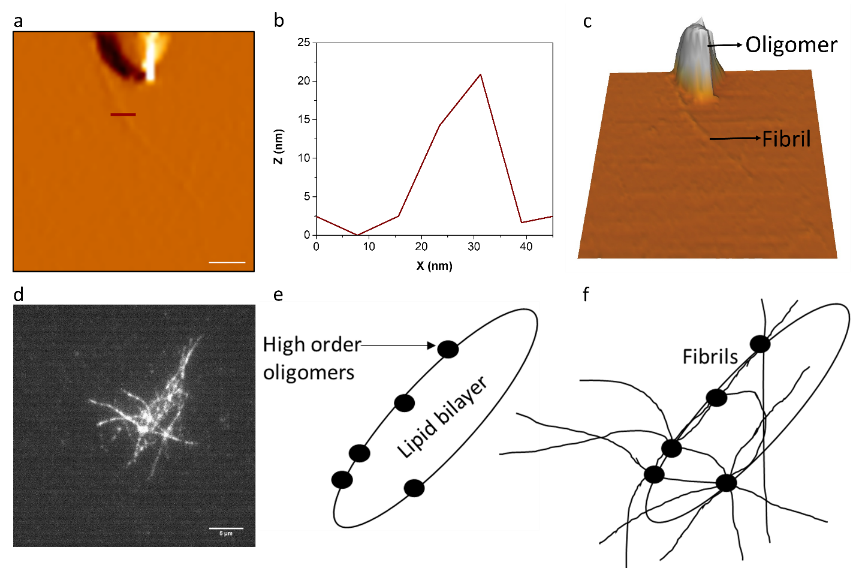


**Supplementary Figure 4: Aβ oligomers may act as seeds for fibril growth on lipid membrane.** (a) AFM topography; (b) Height profile of a mature fibril growing from oligomers across the black section line in (a); (c) 3D image of mature fibril and oligomer; (d) Oligomers with fibril-like structures; (e) Illustration of high order oligomer formation on the edges of lipid bilayers; (f) Illustration of growth of fibrils from high order oligomers. Scale bar in AFM image is 200 nm while in fluorescence image is 5 µm.


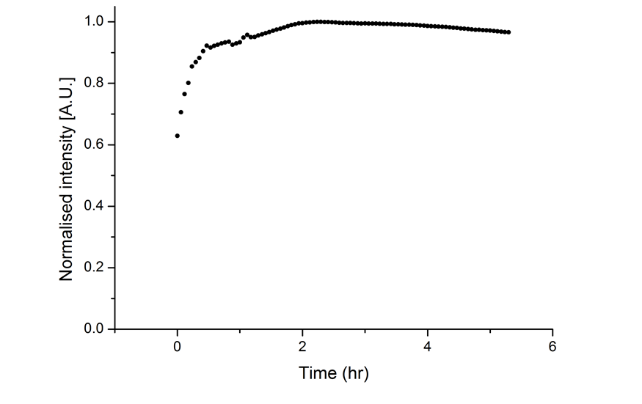


**Supplementary Figure 5: Total fluorescence as a function of time from 100 nM Aβ on DLPC membrane recorded by TIRF imaging. Aβ deposition on the membrane reaches a plateau at 2 hr incubation.**


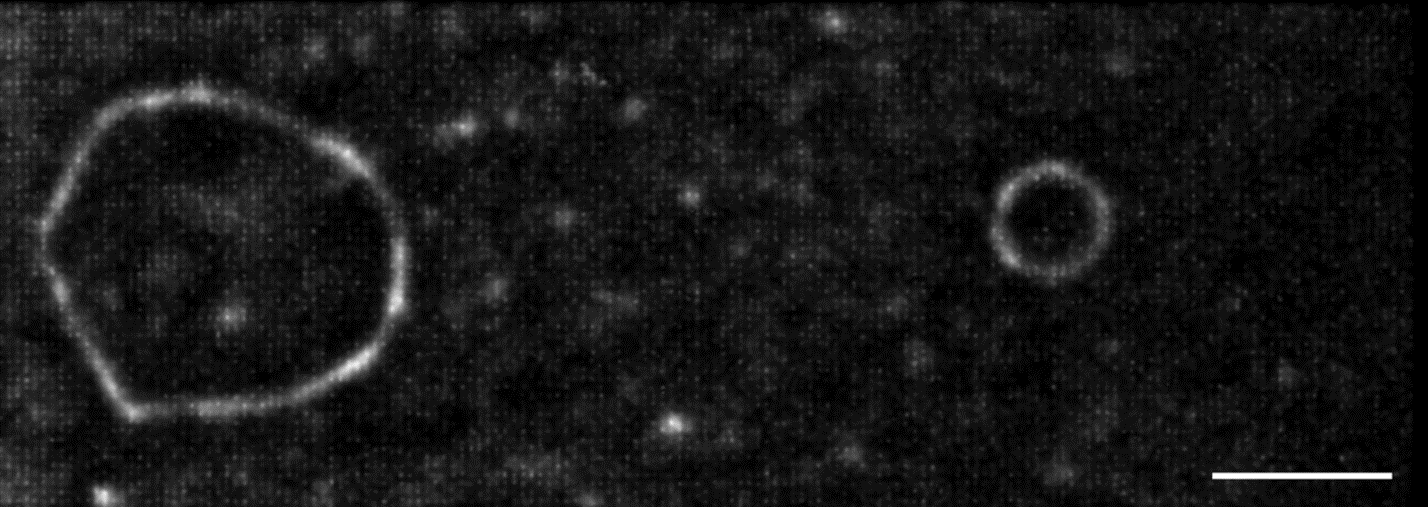


**Supplementary Figure 6: Reconstructed image (probability map) of an annular Aβ assembly.** Fluorescence from labelled Aβ on the DLPC surface was completely photobleached prior to the image sequence acquisition. Single molecule localisations were accumulated from 10000 frames with 15 ms exposure time. Scale bar is 2 µm.


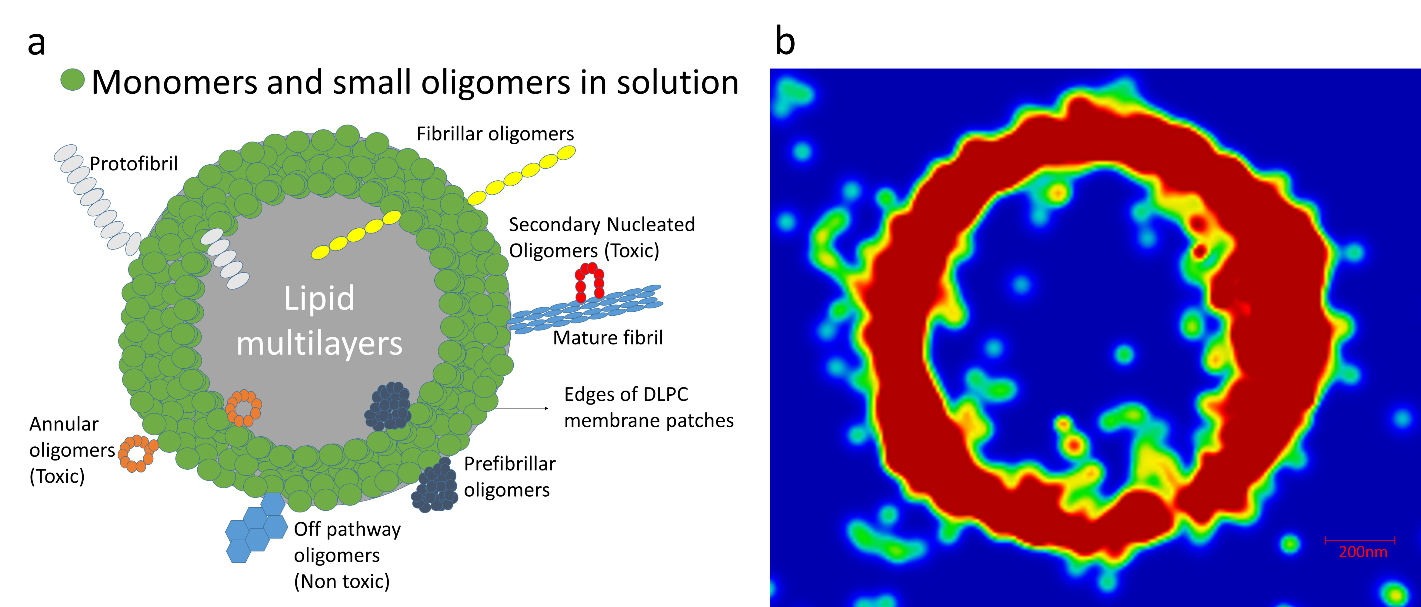


**Supplementary Figure 7: (a) Schematic illustration of Aβ aggregation processes on the edges of a DLPC membrane patch; (b) Coloured reconstructed image by accumulation of single molecules with the superresolution microscope from 10000 frames with 15 ms exposure.** The observation of giant looped oligomeric structures with infinite possibilities of interlinked mechanisms for toxic species formation. Scale bar is 200nm.
